# A survey of retirement intentions of baby boomers: an overview of health, social and economic determinants

**DOI:** 10.1186/1471-2458-14-355

**Published:** 2014-04-14

**Authors:** Anne W Taylor, Rhiannon Pilkington, Helen Feist, Eleonora Dal Grande, Graeme Hugo

**Affiliations:** 1 Population Research & Outcome Studies, Discipline of Medicine, The University of Adelaide, Adelaide, Australia; 2 Australian Population and Migration Research Centre, The University of Adelaide, Adelaide, Australia

**Keywords:** Baby boomers, Retirement, Australia, Survey

## Abstract

**Background:**

Governments have been implementing policies aimed at halting the trend towards early retirement for Baby Boomers. Public policies can have a strong effect on when a person retires and this analysis contributes to an improved understanding of retirement aspirations in regards to health, social, workplace and economic determinants.

**Methods:**

In October 2011 a telephone survey was undertaken with participants aged 50 to 65 years who were in paid employment and who had been in the workforce for the previous three years. Participants were obtained from two identical South Australian cohort studies - the North West Adelaide Health Study and the Florey Adelaide Male Ageing Study. The results of the telephone survey were linked to the original cohort data. Data were weighted by sex, age, postcode and probability of selection in the household. Work related questions included how much they thought about their retirement, current occupation, employment status, type of workplace and hours worked per week. Health related questions included current smoking status, physical activity, body mass index, self-reported health status and overall life satisfaction. Uni-variable and multi-variable analyses were undertaken to compare the different associations between people who were and were not intending to retire.

**Results:**

In total, 25.9% (n = 210) of people who were currently in paid employment indicated that they intend to retire completely from the workforce. The remainder indicated that they will continue to work (41.8% retire from full-time work but work part-time, 25.7% continue working part-time but reduce their current hours, and 6.7% never retire). The multi-variable results indicate that those with lower education, having a savings habit, and sales workers more likely to anticipate complete retirement. The self-employed, and those thinking only moderately about retirement, were more likely to extend their working life beyond age 65.

**Conclusion:**

An important finding of this study is the large number of Baby Boomers who indicated that they would be happy to work part-time or never retire. Policies and continued dialogue aimed at making the workplace a safe, flexible and welcoming environment to accommodate this wish, and to entice others to take up this option over complete withdrawal from the labour force, is required.

## Background

The economic, social and health consequences of Baby Boomers reaching retirement age is a topic of increasing academic and policy interest [1-3]. Baby Boomers, those born between 1946 and 1965, constitute a large proportion of the population of most industrialised countries and has been exacerbated by increased longevity and decreasing birth rates affecting age distributions [4-7]. In Australia, there were 4.2 million babies born between 1946 and 1965 [7] and they now constitute a quarter of the total population [8]. Moreover the post-WWII era was a phase of massive immigration which introduced significant numbers of Baby Boomers born in Europe. In 2011, 33.2% of Australian Baby Boomers were born outside of Australia.

As Baby Boomers start retiring, countries facing this demographic transformation have major concerns. These include retirees outnumbering new entrants to the labour force, loss of skilled and experienced workers [5,9] and the effect on economic growth and income from taxation [10-12]. Coupled with this is the financial burden with increasing numbers requiring tax-funded services such as pensions and other social security benefits [7]. Importantly, the impact on the health system as increased numbers require medical services and intervention is of concern [9,13].

As a result of these apprehensions, governments have been implementing policies aimed at halting the long term trend towards early retirement [7,14-16], contrasting with government policies of the 1970s and 1980s during which early retirement was promoted as a way to lessen the burden of the over-supply of workers [5]. Government initiatives aimed at encouraging Baby Boomers to remain in the workforce, to lessen the effects of an ageing population, include implementing age discrimination laws, removing compulsory retirement age, raising the pension eligibility age and encouraging increased personal saving and planning for retirement [7,17,18]. In addition, policies aimed at extending working life and workplace participation by encouraging a delay or gradual transitions to retirement, and financial incentives for companies who hire older employees have been implemented [9,10]. Notwithstanding these policies, research has shown that early retirement is both voluntary and forced and is influenced by ill-health, lack of job satisfaction and adequate long-term financial resources [9,10,14,19-22].

This study examines a sample of Australian 50–65 year olds and assesses their retirement intentions. As argued by Anderson [6], public policies can have a strong effect on when a person retires and this study aims to contribute to an improved understanding of retirement aspirations in regards to health, social, workplace and economic determinants. Delaying retirement is dependent upon Baby Boomers being willing and able to extend their working life [19] and this research provides an empirical context to these factors to better inform policy making.

## Methods

### Survey design and sample selection

*Australia’s Baby Boomer Generation, Obesity and Work – Patterns, Causes and Implications* is an Australian Research Council (ARC) funded project. As a part of this project, in October 2011 a telephone survey was undertaken with participants from the North West Adelaide Health Study (NWAHS) and the Florey Adelaide Male Ageing Study (FAMAS).

NWAHS commenced in 1999 and is a representative longitudinal cohort study of 4060 randomly selected adults aged 18 years and over at the time of recruitment from the north-west region of Adelaide, the capital of South Australia. Sampling was via the electronic white pages. Major stages of the study have been held approximately every four years and incorporated telephone interviews, self-completed questionnaires and biomedical clinic examinations. A detailed description of the methodology and cohort is available elsewhere [23-25].

FAMAS commenced in 2002 as a longitudinal study assessing the biomedical, socio-demographic, behavioural, physical and psychological interactions that contribute to the health and health-related behaviours of men. The geographical regions, clinic procedures, sampling, recruitment and methodology was identical to NWAHS except it was limited to men aged 35 to 80 years (n = 1195). All major stages of the study have included a self-completed questionnaire and a clinic examination. A detailed description of the methodology and cohort is available elsewhere [26].

A letter and an information sheet introducing the study were sent to each selected participant from the two cohorts. The letter informed participants of the purpose of the study and indicated they could expect to receive a telephone call within the time frame specified. Data were collected by a contracted agency and interviews were undertaken seven days a week. Telephone calls were made between 10.00 am and 8.30 pm. The CATI (Computer Assisted Telephone Interview) system was used to conduct the interviews. Data were collected during October and November 2011. At least 10 and up to 15 call backs were made to each respondent. Different times of the day or evening were scheduled for each call back. If a person could not be interviewed immediately they were rescheduled for interview at a time suitable to them.

A total of n = 1642 interviews were completed, resulting in an overall response rate of 87.5% and a participation rate of 93.0%. For the purpose of this study, analysis was limited to Baby Boomers aged 50 to 65 years who were in the paid employment either full-time, part-time, casual or self-employed and who had been in the workforce for the previous three years (n = 897). The results of the CATI survey were linked to the original cohort data. Data were weighted by sex, age, postcode and probability of selection in the household to the ABS Estimated Residential Population for the NW Adelaide regions for 50–65 year olds.

### Main variables

All respondents who were currently working were asked if they planned to completely retire, retire from full-time work but work part-time, continue to work part-time but reduce hours, or never retire. Respondents were also asked how much they thought about their retirement (a lot, moderately, not at all). Other questions asked in the CATI included highest level of education, marital status, annual household income, financial status (very comfortable, reasonably comfortable, just getting along), household structure (family with child/children, step/blended/sole parent, adult living alone, adults living with partner, adults living together – recoded into living with or without children), and saving habits (which of the following statements comes closest to describing your savings habits? - Don’t save/usually spend more than income; Don’t save/usually spend about as much as income; Save whatever is left over at the end of the month/no regular plan; Spend regular income/save other income; Save regularly by putting money aside each month) with the variable dichotomised into do not save (categories one and two) and save (categories three to five).

Work related questions included current occupation, employment status (full time, part time, casual, self-employed), type of workplace (government, not for profit, private), actual hours worked per week (< 40, 40+), size of workplace, how they felt about their job (satisfied, dissatisfied), job security (poor – agree/disagree), whether it was possible to work from home, whether work interfered with responsibilities and activities outside of work, how satisfied they were with the balance between work and life, and whether their job required a lot of physical exertion.

Health related questions included current smoking status, physical activity (derived on the amount of walking and moderate and vigorous activity in a one week period) [27], body mass index (BMI) which was derived from self-reported weight and height and recoded into three categories (underweight/normal weight, overweight and obese) [28], inadequate daily consumption of vegetables (< 5 serves) and fruit (< 2 serves) [29], number of hours slept per night, self-reported health status (SF1 – excellent/very good, good, fair/poor) and overall life satisfaction. Psychological distress was determined using the Kessler 10 [30,31].

### Statistical analyses

The conventional 5% level was used to determine statistical significance and 95% confidence intervals were provided for estimates. All frequencies and analyses were conducted using weighted data. Uni-variable analyses using chi-square (*χ*^2^) tests were undertaken to compare the different associations between people who were and were not intending to retire, on a range of socio-demographic, socio-economic, health and work-related other variables. Multi-variable analyses using logistic regression modelling was subsequently developed in order to produce a model of the relationship of the independent variables. All independent variables with p values less than 0.25 level in each of the uni-variable analyses were entered into the logistic regression model [32]. Non-significant variables (p > 0.05) based on the log likelihood ratio tests were removed until a final model was obtained.

## Results

Of the eligible respondents (n = 897), 9.4% (n = 84) were excluded from the analyses because they did not know their retirement intention. In total, 25.9% (n = 210) of people aged 50 years and over who were currently working indicated that they intend to retire completely from the workforce with 74.1% (n = 602) indicating that they will continue to work in some capacity (41.8% retire from full-time work but work part-time, 25.7% continue working part-time but reduce their current hours, and 6.7% never retire from workforce).

Table 1 shows the socio-demographic characteristics of the sample (n = 812). Mean age was 56.9 years (standard deviation = 4.48). In total, 80% of the sample were married or living with a partner and over 40% had an annual household income of over $A80,000.

**Table 1:** Demographic characteristics of sample

	**n**	**% (95% CI)**
**Sex**		
Male	407	50.1 (46.6 - 53.5)
Female	406	49.9 (46.5 - 53.4)
**Age**		
50 to 54	305	37.5 (34.3 - 40.9)
55 to 59	266	32.8 (29.6 - 36.1)
60 to 64	241	29.7 (26.7 - 33.0)
**Education**		
Bachelor degree or higher	154	19.0 (16.5 - 21.9)
Certificate/Diploma	185	22.8 (20.1 - 25.8)
Trade/apprenticeship	130	16.1 (13.7 - 18.8)
No schooling to secondary	274	33.7 (30.5 - 37.0)
Not stated/refused	68	8.4 (6.7 - 10.5)
**Marital Status**		
Married/Living with partner	650	80.0 (77.2 - 82.7)
Separated/Divorced	100	12.3 (10.2 - 14.8)
Widowed	27	3.3 (2.3 - 4.8)
Never Married	35	4.3 (3.1 - 6.0)
**Household income per year**		
$80,001+	335	41.2 (37.9 - 44.6)
$40,001-$80,000	275	33.9 (30.7 - 37.2)
$0 - $40,000	133	16.4 (14.0 - 19.1)
Not stated/refused/don't know	69	8.5 (6.8 - 10.6)

Table 2 highlights the uni-variable analysis of demographic factors associated with those who intend to completely retire (as opposed to those who plan to continue to work full or part-time) with females, those with lower levels of education, and those indicating a habit of saving (no regular plan to regularly) being more likely to indicate they would retire completely.

**Table 2:** UNIVARIABLE ANALYSIS of demographic variables associated with the intention to completely retire from the workforce for Baby Boomers aged 50+

	**n/N**	**%**	**OR (95% OR)**	**p value**
**Sex**				
Male	87/407	21.4	1.00	
Female	123/406	30.4	1.61 (1.17 - 2.21)	**0.003**
**Education**				
Bachelor degree or higher	23/154	14.9	1.00	
Certificate/Diploma	40/185	21.4	1.56 (0.88 - 2.74)	0.125
Trade/apprenticeship	35/130	26.7	2.08 (1.16 - 3.75)	**0.015**
No schooling to secondary	95/274	34.8	3.05 (1.83 - 5.07)	**<0.001**
**Marital Status**				
Married or living with partner	166/650	25.6	1.00	
Other	44/162	27.1	1.08 (0.73 - 1.60)	0.692
**Household income per year**				
$80,001+	87/335	26.1	1.00	
$40,001-$80,000	73/275	26.6	1.02 (0.71 - 1.47)	0.903
$0 - $40,000	36/133	26.9	1.04 (0.66 - 1.64)	0.861
Not stated/refused/don't know	14/69	20.1	0.71 (0.38 - 1.35)	0.296
**Given your current financial needs would you say you are…**				
Very comfortable	34/147	23.1	1.00	
Reasonably comfortable	122/473	25.9	1.16 (0.75 - 1.79)	0.502
Just getting along	53/191	27.6	1.27 (0.77 - 2.08)	0.351
**Household structure**				
Parents biological/step/blended/sole with children	57/244	23.2	1.00	
Adults with no children	154/568	27.0	1.22 (0.86 - 1.74)	0.259
**Description of saving habits**				
Don't save	21/121	17.7	1.00	
Save but no regular plan to Save regularly	189/691	27.3	1.75 (1.07 - 2.87)	**0.027**
**Overall**	210/812	25.9		

Table 3 highlights the work related variables with self-employed and those thinking only moderately about retirement being less likely and sale workers, machinery operators and drivers, and labourers more likely to anticipate complete retirement. In addition, those working over 40 hours per week, and those without the possibility of working from home were also more likely to indicate complete retirement.

**Table 3:** UNIVARIABLE ANALYSIS of socio-economic and work related variables associated with the intention to completely retire from the workforce for Baby Boomers aged 50+

**.**	**n/N**	**%**	**OR**	**p value**
**Current employment status^**				
Full-time employee	111/407	27.3	1.00	
Part-time employee	64/208	30.9	1.19 (0.82 - 1.71)	0.359
Casual or temporary	18/56	31.2	1.21 (0.66 - 2.21)	0.541
Self-employed	17/141	12.2	0.37 (0.21 - 0.64)	**<0.001**
**Occupation**				
Managers/Professionals	60/280	21.3	1.00	
Technicians/Trades Workers	18/99	18.4	0.83 (0.46 - 1.49)	0.538
Community and Personal Service Workers/Clerical and Administrative Workers	64/254	25.1	1.24 (0.83 - 1.86)	0.293
Sales Workers	28/63	44.3	2.94 (1.66 - 5.21)	**<0.001**
Machinery Operators and Drivers/Labourers	40/110	36.3	2.11 (1.30 - 3.42)	**0.002**
**Type of workplace**				
Government agency	63/224	27.9	1.00	
A not-for-profit, religious, or community organisation	17/53	31.3	1.18 (0.62 - 2.26)	0.619
A private sector business	130/531	24.4	0.84 (0.59 - 1.19)	0.319
**Size of workplace**				
Small	42/173	24.0	1.00	
Medium	40/151	26.5	1.14 (0.69 - 1.88)	0.613
Large	122/403	30.4	1.38 (0.92 - 2.07)	0.123
**Number of hours work per week**				
40 or less hours	163/558	29.2	1.00	
More than 40 hours	48/254	18.7	0.56 (0.39 - 0.80)	**0.002**
**How much thought about own retirement**				
A lot	124/363	34.1	1.00	
Moderately	59/354	16.7	0.39 (0.27 - 0.55)	**<0.001**
Not at all	28/95	29.1	0.80 (0.49 - 1.30)	0.364
**How much job requires a lot of physical exertion or energy**				
All to some of the time	135/508	26.5	1.00	
Almost none to none of the time	75/304	24.8	0.91 (0.66 - 1.27)	0.587
**General feeling about job as a whole**				
Dissatisfied, not sure	25/108	23.0	1.00	
Satisfied	185/705	26.3	1.19 (0.74 - 1.93)	0.466
**“Job security is/was poor”**				
Agree, not sure	39/151	25.6	1.00	
Disagree	172/661	26.0	1.02 (0.68 - 1.53)	0.925
**Have the possibility to work from home**				
Yes	34/196	17.3	1.00	
No	176/617	28.6	1.92 (1.28 - 2.90)	**0.002**
**Work interferes with [personal] responsibilities & activities**				
Almost always, often, sometimes, rarely	139/575	24.1	1.00	
Never	72/238	30.1	1.36 (0.97 - 1.90)	0.077
**Satisfaction with balance between work and life**				
Satisfied, neither, not sure	178/689	25.9	1.00	
Dissatisfied	32/123	25.9	1.00 (0.65 - 1.55)	0.986
**Overall**	210/812	25.9		

Table 4 highlights the health-related variables with the overweight less likely to anticipate working beyond the retirement age of 65.

**Table 4:** UNIVARIABLE ANALYSIS of health related variables associated with the intention to completely retire from the workforce for Baby Boomers aged 50+

	**n/N**	**%**	**OR**	**p value**
**Self-reported health (SF1)**				
Excellent, Very good	74/326	22.6	1.00	
Good	92/326	28.1	1.34 (0.94 - 1.91)	0.108
Fair, Poor	45/159	28.4	1.36 (0.88 - 2.09)	0.164
**General satisfaction with life**				
Dissatisfied, not sure	29/127	23.1	1.00	
Satisfied	181/685	26.4	1.19 (0.76 - 1.86)	0.442
**Physical activity**				
No activity	40/149	26.6	1.00	
Activity but not sufficient	96/329	29.1	1.13 (0.73 - 1.75)	0.575
Sufficient activity	65/298	21.8	0.77 (0.49 - 1.21)	0.255
**Smoking status**				
Current smoker	32/121	26.5	1.00	
Not a current smoker	163/638	25.5	0.95 (0.61 - 1.48)	0.825
**Daily fruit consumption**				
0 to 1 serves per day	102/421	24.2	1.00	
2+ serves per day (recommendation)	92/326	28.4	1.24 (0.89 - 1.72)	0.203
**Daily vegetable consumption**				
0 to 4 serves per day	186/705	26.4	1.00	
5+ serves per day (recommendation)	8/42	19.2	0.66 (0.30 - 1.45)	0.303
**Psychological Distress**				
No psychological distress	190/742	25.6	1.00	
Psychological distress	21/70	29.3	1.21 (0.70 - 2.07)	0.496
**BMI**				
Underweight, Normal	53/168	31.6	1.00	
Overweight	71/324	21.9	0.61 (0.40 - 0.92)	**0.020**
Obese	62/249	24.9	0.72 (0.47 - 1.11)	0.135
**Total**	210/812	25.9		

Table 5 details the multi-variable results with lower education, saving regularly, and sales workers more likely to anticipate complete retirement and the self-employed and those thinking only moderately about retirement being more likely to extend their working life beyond age 65.

**Table 5:** MULTIVARIABLE ANALYSIS of variables associated with the intention to completely retire from the workforce for Baby Boomers aged 50+

	**OR (95% OR)**	**p value**
**Education**		
Bachelor degree or higher	1.00	
Certificate/Diploma	1.64 (0.89 - 3.02)	0.111
Trade/apprenticeship	2.35 (1.20 - 4.60)	**0.013**
No schooling to secondary	3.08 (1.69 - 5.62)	**<0.001**
**Description of saving habits**		
Don't save	1.00	
Save but no regular plan to Save regularly	1.89 (1.11 - 3.21)	**0.019**
**Current employment status^**		
Full-time employee	1.00	
Part-time employee	0.94 (0.62 - 1.41)	0.752
Casual or temporary	1.11 (0.58 - 2.11)	0.753
Self-employed	0.30 (0.17 - 0.54)	**<0.001**
**Occupation**		
Managers/Professionals	1.00	
Technicians/Trades Workers	0.77 (0.40 - 1.49)	0.438
Community and Personal Service Workers/Clerical and Administrative Workers	0.82 (0.51 - 1.32)	0.414
Sales Workers	2.26 (1.16 - 4.38)	**0.016**
Machinery Operators and Drivers/Labourers	1.51 (0.86 - 2.65)	0.147
**How much thought about own retirement**		
A lot	1.00	
Moderately	0.37 (0.25 - 0.53)	**<0.001**
Not at all	0.89 (0.53 - 1.50)	0.657

## Discussion

This research has identified health, social and economic determinants of Baby Boomers currently working who intend to retire completely, compared to those who are considering continuation of work either in a full-time or part-time capacity. In total, only a quarter of Baby Boomers in this sample planned to retire completely and this analysis has provided a detailed description of this group. As argued by others, the loosening of the retirement definition, as one that is seen as a definitive point in time without ‘shades of grey’, is changing with life course boundaries being loosened [33]. Part-time work, retirement transition and mindsets associated with what constitutes retirement are altering from the traditional, formalised, ‘on-time complete retirement’ concept [20].

Previous research has reported that health has a significant effect on working status in older ages [21,34]. Our measure of overall health was a single self-reported question (SF1), which has been shown to be a powerful predictor of actual health status [13,35]. This measure was not significant at the uni-variable or multivariable stage, indicating no difference in health status between those intending to retire completely at age 65 (the Australian government current recommended retirement age) and those planning to continue to work. It should be noted that our analysis was limited to those currently in the work force and respondents who were unemployed or stated home duties as their current work force status were excluded and may indeed have poorer health status. An Australian study has also shown that reducing risk factors increases labour force participation [36] but our study has shown that there is little relationship in our study between risk factors, as measured by physical activity, smoking status, fruit and vegetable consumption and BMI, and Baby Boomers plans to retire, especially at the multi-variable level.

Only one of three financial and economic related variables included in our analysis (household income, financial security and ability to save) were included in the multi-variable model with those indicating that they had a saving habit nearly twice as likely to indicate a preference to retire completely. It should be noted that no measure of wealth *per se* was assessed and this could be a confounding variable. Previous research has shown one of the major contemporary reasons for extending working life is lack of financial security [3,22]. The Global Financial Crisis (GFC) originating in 2008, has affected all developed and developing countries [37] and in particular many of the current wave of Baby Boomers [18,37,38]. Previous Australian qualitative research conducted in 2008 reported that the majority of workers aged 50 to 62 years were re-assessing their retirement plans as a result of the GFC [38]. Research undertaken in 2009 reported that nearly 40% of workers aged 50 to 64 years had postponed their retirement plans [18] and other commentators have also suggested that Baby Boomers are increasingly staying in their jobs and that the impact of the GFC might be part of their decision making process [39]. Our results perhaps indicate that this hesitation in retiring completely has continued and the popular stereotype that Baby Boomers are not savers and are not saving for their retirement needs to be challenged [40].

Education level has previously been shown to not be related to intention to retire [20] although our multi-variable analysis shows that the more highly educated are more likely to continue to work part time or to never retire. As argued by others [20] education is also perhaps confounded by income, wealth, job security and job flexibility. Education attainment is also reflected in occupation. Other studies have reported similar results with the higher educated more likely to continue to work [15].

Another major finding of the study was the association between occupation and retirement intentions, with sales workers more likely to indicate full retirement as their choice. Research has shown that those in physically demanding jobs are more likely to retire early and completely [16,41,42]. In recent decades structural economic change has seen physically demanding jobs decrease although at the same time there has been an increase in high-stress jobs and cognitive challenging jobs which can also influence retirement plans [16]. Middle aged females are critical to the labour supply and are seen as a key policy target. Many of these are in the sales industry. Recent labour force trends show that more women aged 55 years and over are remaining in the work force than previously [39] and the most marked change was in the older 60 to 64 year age group. A large part of these increases are of part-time workers although those in full-time jobs have also increased. While not assessed in this analysis, the ‘dual issues’ of the formal carer shortage, and the current caring role of many Baby Boomers (limiting their participation in the work force), makes balancing the ‘caring’ role with work, a key policy issue. The caring responsibilities of mid-life women may impact on employment roles and pathways to increase workforce participation opportunities for this group warrants serious consideration [14,15].

The self-employed were less likely to report retirement as an option. The retirement intentions of the self-employed have not been extensively researched [42,43] although research has highlighted the average age is higher for self-employed retire than those employed by others [42]. It has been suggested that one self-reported group, who typically have been self-employed for a longer period of time, appreciate the flexibility, security and autonomy associated with being self-employed. A higher income around the retirement age also decreases the likelihood of permanently retiring. For a second group, the lack of financial security is a possible reason for the lack of retirement plans. It is apparent that self-employment for this second group is not a transitional process on the path to full retirement with research showing that these self-employed older workers are more likely to have had many short term and part-time positions and poor job prospects [42,43].

The major weakness of this study is the self-reported nature of the data collection, common in all survey research methodology. Additional clarification on timeframe for retirement intension and the extent of how they were thinking about retirement would have been beneficial. Strengths include the relatively large sample size and the large number of variables assessed.

## Conclusion

This research has highlighted a range of attributes associated with Baby Boomers planning to retire completely and reinforces the need for policies to encourage extension of working lives need to take account of a range of factors. There are no single ‘silver bullet’ solutions [44]. While acknowledging that some issues, such as work conditions, can be influenced by appropriate policy interventions, other factors, such as education, are not amenable to policy. However, increased flexibility with working hours may overcome some of these harder to influence factors [44]. The flexibility of working arrangements is an important policy [45] so that work-life balance is not compromised. The culture of early retirement, previously so pervasive, is being replaced by a culture of gradual retirement, encompassing continued part-time employment [1]. An important finding of this study is the large number of Baby Boomers who will be happy to work part-time or never retire. Policies and continued dialogue aimed at making the workplace a safe, flexible and welcoming environment to accommodate this wish, for this age group, is required.

## Abbreviations

ARC: Australian research council; BMI: Body mass index; CATI: Computer assisted telephone interviewing; FAMAS: Florey adelaide males ageing study; GFC: Global financial crisis; NWAHS: North west adelaide health study.

## Competing interests

The authors declare that they have no competing interests.

## Authors’ contributions

AT – Conception and design, interpretation of data, drafting manuscript. RP – Analysis and interpretation of data. HF – Interpretation of data. EDG – Interpretation of data. GH - Conception and design, interpretation of data. All authors have reviewed the manuscript critically for important intellectual content. All authors have given final approval of this version to be published.

## Pre-publication history

The pre-publication history for this paper can be accessed here:

http://www.biomedcentral.com/1471-2458/14/355/prepub
